# Atypical histological presentation of bone regeneration after insertion of cryoprotected allogeneic bone graft

**DOI:** 10.4317/medoral.26094

**Published:** 2023-11-22

**Authors:** Pamela Cruz, Joao De Bortoli, Ernesto B Benalcázar-Jalkh, Suheil M Boutros, Monish Bhola, Federico Grande, Vasudev V Nayak, Nick Tovar, Paulo G Coelho, Lukasz Witek

**Affiliations:** 1 BS. Biomaterials Division - New York University College of Dentistry, New York, NY, USA; 2 DDS PhD. Biomaterials Division - New York University College of Dentistry, New York, NY, USA; 3 DDS, MSci, PhD. Department of Prosthodontics and Periodontology, University of Sao Paulo, Bauru School of Dentistry, Bauru, SP, Brazil; 4 DDS, MS. Private practice Grand Blanc, Flint and Clarkston, MI.; Adjunct Clinical Assistant Professor of Dentistry, Department of Oral Medicine and Periodontics, University of Michigan, Ann Arbor, MI, USA; 5 BDS, DDS, MSD Director. Advanced Periodontics Institute and private practice, Livonia, MI, USA; 6 DDS, MD. Private practice, Stuart, Florida, USA; 7 MSci, PhD. Department of Biochemistry and Molecular Biology, University of Miami Miller School of Medicine, Miami, FL, USA; 8 PhD, DDS. Biomaterials Division - New York University College of Dentistry, New York, NY USA; Department of Oral and Maxillofacial Surgery, New York University, Langone Medical Center and Bellevue Hospital Center, New York, NY, USA; 9 MD, DDS, PhD, MBA. Division of Plastic Surgery, Department of Surgery, University of Miami Miller School of Medicine, Miami, FL, USA; Department of Biochemistry and Molecular Biology, University of Miami Miller School of Medicine, Miami, FL, USA; 10 MSci, PhD. Biomaterials Division - New York University College of Dentistry, New York, NY USA; Hansjörg Wyss Department of Plastic Surgery, NYU Grossman School of Medicine, New York, NY USA; Department of Biomedical Engineering, Tandon School of Engineering New York University Brooklyn, NY USA

## Abstract

**Background:**

To evaluate bone regenerative capacity of cryoprotected corticocancellous allogeneic bone graft performed in type II and III post-extraction sockets for ridge preservation after twelve weeks in-vivo.

**Material and Methods:**

Twenty-seven type II or III bony-walled extraction sockets (mandible and maxilla) were selected for this study. Following atraumatic tooth-extraction a cryoprotected corticocancellous allogeneic bone graft material and a resorbable porcine-derived collagen membrane were used for ridge preservation. During re-entry surgery at approximately 12 weeks, bone core biopsies were obtained using a 3.2 mm trephine drill and samples were histologically processed and subjected to qualitative and quantitative histomorphometric analysis. Quantitative data was analyzed using a general linear mixed model with results presented as mean values with the corresponding 95% confidence interval values.

**Results:**

Healing without incident and ridge preservation allowed for the placement of dental implants after 12 weeks in 25 out of the 27 treated socket sites. Analyses yielded an average of ~21.0±7% of old/native bone, ~17±5.5% of newly regenerated bone (total of ~38±12.8% for all bone), 0.23±0.14% of new bone presenting with nucleating sites within the matrix, ~52±5.12% of soft tissue, and 3.6±2.09% of damaged bone. The average regenerated bone was statistically analogous to that of old/native bone (*p*=0.355). Furthermore, an atypical histological pattern of bone regeneration was observed, with newly formed bone exhibiting “infiltration-like” behavior and with new bone nucleating sites observed within the demineralized bone matrix.

**Conclusions:**

Cryoprotected corticocancellous allogeneic bone-graft demonstrated osteoconductive, osteoinductive, and osteogenic properties, yielding unique healing patterns which does warrant further investigation.

** Key words:**Bone regeneration, bone graft, allograft, ridge preservation, dental implant.

## Introduction

Reconstructive techniques in maxillofacial surgery commonly utilize bone grafts to rehabilitate osseous tissue following loss due to trauma, infection, congenital disease, surgically created osteotomies, or tooth extraction (1). Variations in morphologies of alveolar ridges following tooth loss have been reported to yield functional and esthetic defects that may compromise the prognosis of dental implant treatment (2). As a result, the technique of ridge preservation was developed to preserve the alveolar dimensions during the healing phase after tooth extraction (3).

Ridge preservation and bone grafting procedures, the second most common transplantation following blood transfusion, have generated favorable outcomes (4). The utilization of bone grafting materials in combination with dental implants is a common treatment modality in oral and maxillofacial reconstructive surgery. There are a variety of grafting materials commercially available, offering surgeons alternatives for various procedures (1). Contingent on the anatomical location of a procedure, the selected bone substitute should possess appropriate characteristics for bone healing/regeneration capacity and mechanical support (5). For ridge preservation techniques, the graft serves as a beneficial resource to minimize alveolar ridge resorption by providing a scaffold for bone regeneration (6).

Alveolar ridge preservation procedures are accomplished/performed by using natural (non-synthetic) materials such as autogenous, allogeneic, and xenogeneic grafts, or synthetic biomaterials such as ceramics, metals, polymers, and composites (7). The selection of material significantly depends on the quality and presence of osteoconductive, osteoinductive, and osteogenic properties (8,9).

Autogenous bone grafts remain the gold standard for bone regenerative procedures (10,11). Autogenous bone possesses the properties required for osteoconduction, osteoinduction, and osteogenesis mechanisms for bone healing and regeneration. Despite these important properties, limitations such as the need for secondary surgical site to harvest the graft, significant donor site morbidity, limitations in quantity, among potential complications have led to the development and study of alternative grafting materials (12).

Alternative bone grafting materials include xenografts, allografts and alloplasts. Xenogeneic grafts are obtained from a different species. Bovine-derived bone substitute, for example, is commonly utilized in the oral and maxillofacial surgical field. The graft possesses natural hydroxyapatite (HA) and inorganic bone matrix, which serve as a microscopic scaffold enhancing bone healing and regeneration. Although it offers appropriate osteoconductive properties, xenografts are associated with a slow remodeling process, accompanied with the resorption of the graft material and the mechanical properties of the new bone formed (13). Allogeneic grafts are harvested from the same species as the recipient, commonly cadaveric bone (4). Similar to xenografts, these can induce an immunological response. Recent advances in chemical and physical processes have successfully removed any pathogenic properties (decellularization) that could otherwise compromise the recipient’s immune system (10).

Other important elements of allografts are the osseous composition and the preservation protocol. These grafts are commercially available in cancellous, demineralized cortical, or a cancellous-cortical mixture form. The grafts can be prepared as either fresh-frozen bone (FFB) or freeze-dried bone (FDB) (14). The osteoconductive properties of cancellous bone provide an interconnected trabecular structure that facilitates bone in-growth, and graft remodeling. Additionally, the osteoinductive properties of demineralized cortical bone fibers provide; 1) bone morphogenetic proteins (BMP-2, 4 and 7) which have been shown to trigger the differentiation and proliferation of bone-forming cells; and 2) inherent growth factors (TGF-ß1, VEGF and IGF-1) which are known to support several other aspects of the bone-formation process (15,16). Osteogenic properties naturally present in cancellous bone provide viable cells such as mesenchymal stem cells, osteoprogenitor cells as well as pre-osteoblasts that lead to bone formation and the fusion process of the new osseous tissue (4,8).

Specialized processing protocols allow for preservation of important qualities of the harvested bone. For example, the retention of naturally adherent osteogenic cells of cancellous bone. Such preservation is accomplished under cryopreserve conditions (-70°C), whereas the grafting material is frozen via a controlled process using dimethyl sulfoxide (DMSO) and glycerol, removing the water crystals formed during the freezing process that have the potential to otherwise damage viable cells (17). This preservation method can successfully recover 80% to 95% of the cells while maintaining their biological qualities after the thawing (17,18).

Cryoprotected corticocancellous allogeneic bone matrix (VCBM) graft material is procured from human tissue donors, complying with all regulatory suitability requirements, and processed by tissue banks accredited by the American Association of Tissue Bank (AATB) (19). Following processing, the resulting cortical demineralized bone matrix fibers and cancellous chips containing osteogenic cells are combined in a 1:1 ratio, respectively and frozen at -70°C in a cryoprotective solution in an effort to preserve cell viability and biological properties of the grafting material (20). This study presents the characterization of an allogenic VCBM grafting material (known commercially as PrimaGen Advanced™ Allograft, Zimmer Biomet Spine, Inc., Westminster, Colorado, USA) and a pilot prospective clinical trial that aimed to evaluate its bone regenerative capacity for ridge preservation in type II and III post-extraction sockets after twelve weeks in-vivo.

## Material and Methods

- Scanning Electron Microscope (SEM) characterization

SEM imaging was performed to qualitatively assess the morphology of the DBM fibers and mineralized particulates of PrimaGen AdvancedTM Allograft (Zimmer Spine, Inc., Westminster, Colorado, USA). After storage at -70°C, 10% formalin solution was added to the frozen sample to fix the samples, which were subsquenelty stored at 4°C for 24 hours to thaw the tissue simultaneously with fixing. A second round of fixation was performed with 2.5% glutaraldehyde for 15 minutes and the sample was washed three times with 1x PBS for 5 minutes per wash. Following the wash, the dehydration process was performed in series of ethanol solutions (50%, 75%, 95%, 2x 100 % in DI-H2O), followed by series of HMDS (25%, 50%, 75%, 100% in EtOH), for 15 minutes each. Finally, the sample was left in HMDS overnight (~12 hours) in order to allow for the gradual evaporation of HDMS and complete drying of the tissue.

Once prepared the samples were gold coated and subjected to SEM imaging at 10.0 kV to evaluate the morphology of DBM fibers and to evaluate the surface of the mineralized cancellous particles.

- Clinical trial design, eligibility criteria and ethical considerations

This was a prospective, three-center, non-randomized, pilot clinical study. Enrollment was open to all male and female patients of at least 18 years of age requiring dental implant therapy. Patients eligible for enrollment in the study were those presenting a 2 or 3-walled bony defect in at least one extracted tooth site (Type II and III sockets only) and requiring bone grafting to support a dental implant.

The clinical study was conducted in accordance with the the International Conference on Harmonisation - Good Clinical Practices, and applicable regulatory requirements in agreement with the Declaration of Helsinki regarding the Ethical Principles for Medical Research Involving Human Subjects. Approval from an independent Institutional Review Board (WIRB - Western Institutional Review Board, Puyallup, WA) was obtained (approval # 20172666) and all patients enrolled in the study provided informed consent to participate. Additionally, study was conducted following the ethical principles founded in the declaration of Helsinki. The Consolidated Standards of Reporting Trials (CONSORT) guidelines were used as the framework for this study and report. All recognized regulations for clinical trials involving human subjects, including but not limited to the ICH (International Conference on Harmonization) guidance for industry- E6 Good Clinical Practice: Consolidated Guideline, and the Declaration of Helsinki; FDA regulations on research with human beings (21 CFR 50 and 56); the Health and Human Services (HHS) Regulations on research with human beings (45 CFR 46 Subparts A, B, C, and D); the Health Insurance Portability and Accountability Act of 1996, were strictly followed during the conduct of this study. Before the start of the study, all potentially eligible patients were present with the protocols aims, methods, anticipated benefits, and possible hazards that their participation can stimulate as well as were provided with the written description of the study protocol. All patients meeting protocol inclusion criteria were asked to sign an informed consent form prior to receiving any study-related treatment

Twenty-seven type II or III bony-walled extraction sockets (mandible and maxilla) in twenty-three patients (11 males, 12 females aged 24 to 82 years; mean age: 54) were included in this study across the three private practice clinics (USA). Exclusion criteria for enrollment in the study included patients with uncontrolled systemic disorders, active infection or severe inflammation at the treatment site, current users of non-steroidal anti-inflammatory drugs, bisphosphonates or corticosteroid treatments, those with severe parafunctional habits (such as bruxism or clenching), smokers (more than 10 cigarettes per day), those with a history of therapeutic radiation to the head or jaw, having an active HIV or Hepatitis infection, and women who were pregnant.

Complete information regarding treatment, materials to be used, and follow-up appointments was provided to the patients, who provided informed consent to participate of the study. Patients did not receive a monetary incentive and were not participating of any other clinical research study. Patients returned to respective clinic for a total of six visits - one screening visit to obtain medical history and dental assessments; one for tooth extraction and grafting procedure; three healing assessments (7-10 days, 4 weeks, and 8 weeks); and a final visit for re-entry surgery, bone biopsy retrieval and implant placement.

- Surgical procedures

After administration of local anesthesia, the tooth was atraumatically extracted using full thickness mucperiosteal flaps technique with less than 3mm elevation from the socket crest. The socket was thoroughly debrided with surgical curettes and rinsed with saline solution.

All patients received bone graft (PrimaGen AdvancedTM Allograft, Zimmer Spine, Inc., Westminster, Colorado, USA) that was thawed and hydrated following instructions provided in the Instructions for Use (IFU). The material was incrementally packed into the socket until it reached the bony crest of the extraction site. Grafted sites were then covered with a resorbable porcine-derived collagen membrane (CopiOs® Extend membrane, Zimmer Biomet, LLC, Palm Beach Gardens, FL) and sutured with 4.0 PTFE sutures. Primary closure was not attempted, but no more than 3 mm of the membrane was left exposed beyond the bony wall of the dehiscence.

All patients received an antibiotic regimen of Clyndamicin taken 4 times a day for 7 days. Patients were also prescribed analgesics for pain and antimicrobial rinse for one week and were instructed to follow a semi-liquid diet for 48 hours following the surgery. The patients returned for postoperative assessment at 7 - 10 days, 4 weeks and at 8 weeks after the procedure.

Following an approximate healing period of 3 months, patients returned for implant placement surgery where clinical measurements were repeated prior to osteotomy preparation. At this time, bone core biopsies were harvested using a 3.2 mm trephine drill (Zimmer Biomet Dental). The bone core samples were fixed in a 10% formalin solution for subsequent histological preparation and analysis. The cored sites received a dental implant (T3 with DCD Platform Switched Implants and Tapered Screw-Vent® with full MTX surface texturing and microgrooves, Zimmer Biomet Dental) placed according to implant manufacturer’s recommendations.

- Histological Preparation and Histomorphometrical analyses

The surgically removed tissue biopsies were gradually dehydrated in a series (70-100%) of ethanol solutions and then embedded in a methyl methacrylate based resin. Embedded blocks were cut into thin sections (~250μm) using a low speed saw equipped with a diamond saw (Isomet 2000, Buehler Ltd., Lake Bluff, IL). The sections were glued to slides and ground on a grinding machine (Metaserv 3000, Buehler, Lake Bluff, IL) under water irrigation with a series of SiC abrasive paper (Buehler, Lake Bluff) until they were approximately 100m thick. The samples were stained in Stevenel’s blue and Van Geison to differentiate the soft and connective tissues. Samples were quantitatively analyzed using histology micrographs and image analysis software (ImageJ, NIH, Bethesda, MD). Fraction occupancy (FO) of native bone, new bone, and soft tissue was quantiﬁed to analyze the osseo regeneration parameters in combination with the demineralized bone matrix. Quantitative analyses were performed by a calibrated single blind evaluator after a good intraclass correlation coefficient (between 0.9 to 1) was obtained in the intra-rater reliability measurements. All data is reported as a function of percentage (21).

- Statistical Analysis

All histomorphometric data are presented as mean values with the corresponding 95% confidence interval values (mean ± 95% CI). Prior to statistical analysis a normality test was performed after which the data was analyzed using a linear mixed model. All analyses were completed with IBM SPSS (v25, IBM Corp., Armonk, NY). The methodology of the present study was reviewed by an independent statistician.

## Results

Results were reviewed by an independent statistician.

**Figure 1:**
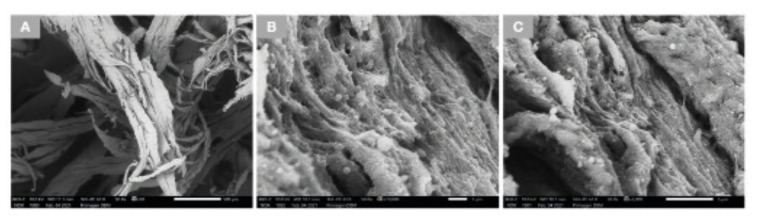
SEM images of DBM denote the presence of fibers with various lengths and thicknesses (A). Higher magnification images (B and C) exhibit the surface morphology of individual fibers.

**Figure 2:**
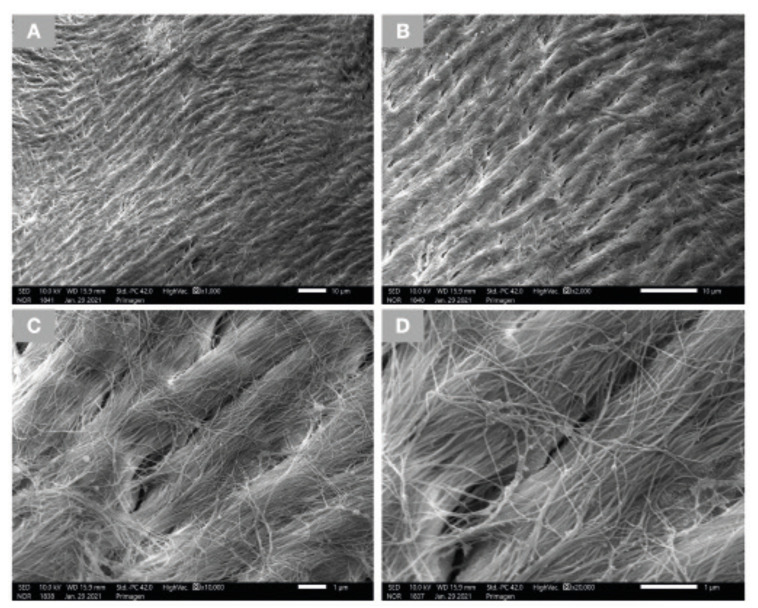
SEM images of mineralized particulates denote the microarchitecture of bone. Figures A and B demonstrate the preservation of the mineralized collagen compartment. Figures C and D evidence the presence of unorganized fibers on the surface of the particulates, speculated to be remnants of endosteum or another extracellular matrix.

Low magnification SEM micrographs of the DBM (Fig. 1), revealed the presence of multiple fibers of various lengths and thicknesses. Higher magnification micrographs (Fig. 1) reveal the surface morphology of individual fibers. Additionally, imaging of the mineralized particulates revealed that the mineralized collagen compartment of the bone structure was preserved after processing without any noticeable damage to its microarchitecture (Fig. 2). High magnification SEM images indicate the presence of fibers on the surface of the particulates, which are not organized in a bundle form similar to those seen in lower magnifications and are speculated to be remnants of endosteum or another extracellular matrix.

The clinical trial constituted enrollment of a total of twenty-three patients (with 27 treated sites) requiring ridge preservation. Two patients did not complete the study successfully. One of the patients developed an infection at the treated site, requiring additional healing time and further interventions. The second patient did not comply with subsequent study visits per protocol. Thus, a total of 25 bone core biopsies were used for analysis.

Some of the adverse events reported were swelling at the site, sinus perforation, and loss of taste. There were no serious adverse events reported. A summary of treated regions, socket type is provided in Table 1.

At the re-entry surgery, all treated sites were reported to depict new bone formation that permitted for a conventional osteotomy and subsequent dental implant placement with average torque no greater than 35 N⋅cm.

**Figure 3:**
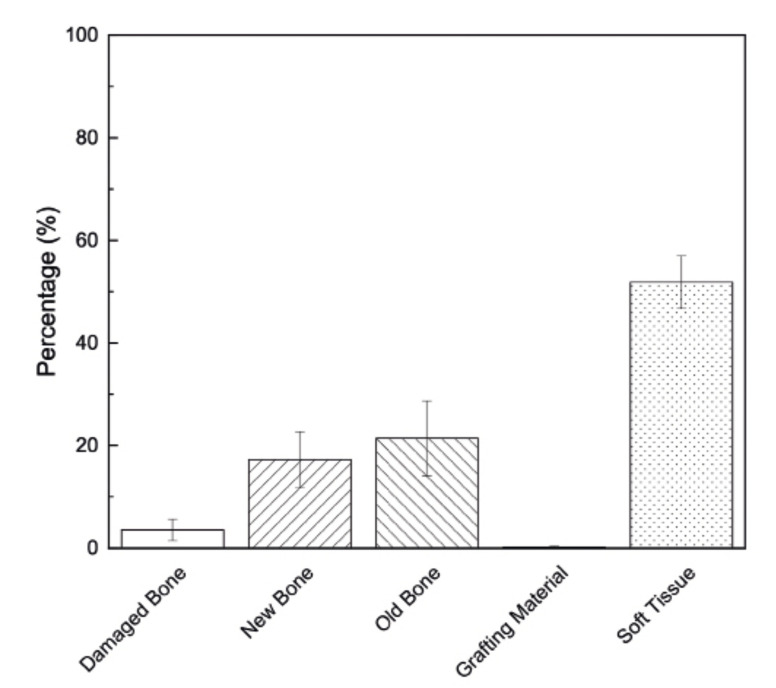
Statistical analysis presenting the average percent and 95% confidence intervals of all the elements part of the histomorphometrical analysis.

Histomorphometrical analysis yielded an average of 21±7.32% of old/native bone, 17±5.47% of new bone (resulting a total bone of ~38±13%), 0.23±0.14% of newly regenerated bone nucleating sites within the matrix, 52±5.12% soft tissue, and 3.6±2.09% damaged bone. Statistical analysis detected the average of new bone was statistically analogous to that of old bone (*p*=0.355) (Fig. 3). Histological analysis quantified lamellar bone as new bone.

**Table 1:**
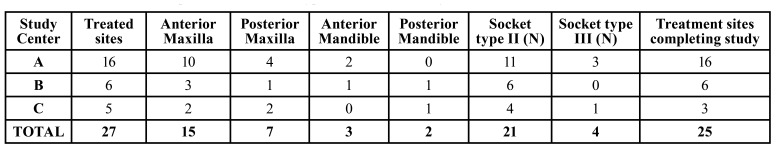
Treated tooth sites and post-extraction socket types included in the study.

**Figure 4:**
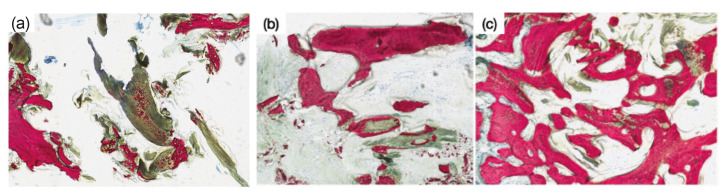
(a) Histological micrographs evidenced the presence of osteoblasts and lacunae, which permitted for the differentiation of newly formed bone from the old osseous tissue; (b and c). Newly formed osseous tissue nucleating sites were observed within the DBM. Such infiltration-like behavior gives the appearance of “particles” within the demineralized bone matrix.

Histological micrographs from each sample confirmed the presence of osteoblasts and lacunae, which permitted for the differentiation of newly formed osseous tissue from the old osseous tissue (Fig. 4). Further surveying of the micrographs revealed the presence of the grafting material (Fig. 4) with atypical histological behavior of bone regeneration. It is noteworthy that newly formed osseous tissue nucleating sites were observed within the demineralized bone matrix instead of surrounding the material. Such observation indicates that the newly formed osseous tissue had an infiltration-like behavior, giving the appearance of “particles” within the demineralized bone matrix (Fig. 4).

## Discussion

It has been well established that there are two primary bone healing mechanisms: direct bone growth and indirect bone growth after callous formation (5). The first involves the development of bone from the broken ends of the fracture site without any intermediate fibrous tissue formation. The latter involves inflammation leading to callous formation via intramembranous ossification followed by endochondral ossification and resorption of the callous (22). The healing processes encompass osteogenic progenitor cells, which are recruited by various cytokines and growth factors (23). These cells actively contribute to osseointegration and bone repair. The timing and concentration of these cytokines and growth factors are critical considerations in designing biomaterial scaffolds.

Alveolar ridge preservation following tooth extraction may be accomplished through either of the aforementioned bone healing mechanisms, where best results allow the maintenance of horizontal and vertical dimensions of the ridge following a healing time of 3 to 4 months for bone consolidation. Advantages of ridge preservation include reducing the early alveolar bone loss following tooth extraction, whereas disadvantages include the inability to gain more height or width than tooth socket size and slow bone remodeling of graft material. Furthermore, common complications include graft infection, loss of graft material, and loss of alveolar ridge width or height (6).

Although a blood clot may be sufficient for the onset of the healing process, fresh frozen bone (FFB) does lend itself to be an effective additive for ridge preservation. Using allografts eliminates donor site morbidity, and ideally minimizes ridge resorption in comparison to a non-preserved extraction socket (24,25). Previous research has demonstrated that FFB can maintain cellular vitality despite being subjected to the freezing process (-80°C) and is viable even when stored for six months in a bone bank (26). Osteoblast-related cells can be grown *in vitro* from FFB specimens, using cells derived from frozen grafts that are morphologically indistinguishable from those grown from freshly harvested trabecular bone (26).

For biologic reconstruction to be achievable, osseous tissue must be preserved and stored for extended periods. However, some freezing protocols may damage cells and degrade the graft material's osteogenic potential *in vivo* (27). Literature has shown high survival rates for implants placed in ridges regenerated with FFB, similar to mandibles grafted with autologous iliac crest bone, which suggests FFB be a reliable grafting substitute restoring mandibular alveolar ridges (28).

A recent systematic review and meta-analysis on ridge preservation techniques following tooth extraction, suggests that particulate xenogeneic or allogenic graft covered with a resorbable membrane is most favorable treatment (29). This finding is evident from the results of the present study, as no signs of graft rejection were observed. Furthermore, all treated sites in the study healed uneventfully and allowed for the placement of dental implants. Advantages of allografts include availability, size and shape, and the elimination of secondary surgical site surgery (30,31). However, potential disease transmission from the donor to the recipient, higher absorption rate, decreased revascularization, and immunogenic response remain a viable concern (32). The allograft used in this study undergoes rigorous screening, testing and disinfection processes to minimize potential disease transmission and infection. SEM imaging in the present study evidenced the preservation of DBM fibers and the mineralized collagen compartments within the grafting material. Since microarchitecture is of paramount importance for the biological and mechanical properties of the graft, its preservation through processing and storage is highly desirable (31).

Histological analysis of the interface between allograft and newly formed osteoid has demonstrated a direct interaction between the allograft and the recipient osteoblasts, which lined the surface of dead bone and appositionally deposited new bone (33). The findings of the present study suggest that cryoprotected cortico-cancellous allogeneic bone graft for alveolar ridge preservation, resulted in increased new bone formation and high percentage of total bone (new and old) after three months *in vivo*. More importantly, there was an increased presence of osteoblast and an atypical histological presentation of bone regeneration, with newly formed osseous tissue exhibiting infiltration-like behavior and displaying new bone nucleating sites within the demineralized bone matrix. Although the results demonstrate that the allograft has adequate osteoconductive, osteoinductive, and osteogenic properties, the histological findings of the study appear to be unique. These characteristics have not been observed with other graft materials and merit investigations. Furthermore, the interpretation of the present findings should consider the reduced number of samples and the absence of randomization as the main limitations of the present pilot study. Complete randomized clinical trials including different grafting materials for alveolar ridge preservation are warranted.

Overall improvement in bone grafting technology continues to be relevant in present times. Tissue engineering approaches use porous implant materials, such as calcium phosphate ceramics, as carriers for bone cells and osteoinductive molecules. Mesenchymal cells (MSCs) have been widely used in this application because they can differentiate to bone-forming cells (osteoblasts) and promote fracture healing. The literature indicates that seeding porous biomaterial matrix with cryopreserved MSCs and using this matrix as a bone graft is a promising answer to bone reconstruction. Therefore, cryopreservation of cellular material can maintain the osteogenic capabilities of stem cells and provide easy access to such types of bone graft materials for cranio-facial and maxillofacial applications (34).

## Conclusions

Cryoprotected cortico-cancellous allogeneic bone-graft resulted in a high presence of osteoblast and an infiltration-like behavior of newly formed osseous tissue, with new bone nucleating sites present within the demineralized bone matrix. This atypical histological presentation of bone regeneration observed in the present pilot study suggests favorable osteoconductive, osteoinductive, and osteogenic properties of the allogeneic bone-graft, while the different healing pattern warrants further investigation.
